# Fertilisation and early developmental barriers to hybridisation in field crickets

**DOI:** 10.1186/1471-2148-13-43

**Published:** 2013-02-15

**Authors:** Frances Tyler, Rolando Rodríguez-Muñoz, Tom Tregenza

**Affiliations:** 1 Centre for Ecology and Conservation, College of Life and Environmental Sciences, University of Exeter, Cornwall Campus, Penryn, Cornwall, UK

**Keywords:** Speciation, Reproductive isolation, Hybrid, Fertilisation, Embryogenesis, Gryllus, Variable reproductive isolation, Polymorphism

## Abstract

**Background:**

Post-mating interactions between the reproductive traits and gametes of mating individuals and among their genes within zygotes are invariably complex, providing multiple opportunities for reproduction to go awry. These interactions have the potential to act as barriers to gene flow between species, and may be important in the process of speciation. There are multiple post-mating barriers to interbreeding between the hybridising field crickets *Gryllus bimaculatus* and *G. campestris.* Female *G. bimaculatus* preferentially store sperm from conspecific males when mated to both conspecific and heterospecific partners. Additionally, conspecific males sire an even greater proportion of offspring than would be predicted from their sperm’s representation in the spermatheca. The nature of these post-sperm-storage barriers to hybridisation are unknown. We use a fluorescent staining technique to determine whether barriers occur prior to, or during embryo development.

**Results:**

We show that eggs laid by *G. bimaculatus* females mated to *G. campestris* males are less likely to begin embryogenesis than eggs from conspecific mating pairs. Of the eggs that are successfully fertilised and start to develop, those from heterospecific mating pairs are more likely to arrest early, prior to blastoderm formation. We find evidence for bimodal variation among egg clutches in the number of developing embryos that subsequently arrest, indicating that there is genetic variation for incompatibility between mating individuals. In contrast to the pattern of early embryonic mortality, those hybrids reaching advanced stages of embryogenesis have survival rates equal to that of embryos from conspecific mating pairs.

**Conclusions:**

Post-sperm-storage barriers to hybridisation show evidence of genetic polymorphism. They are sufficiently large, that if the species interbreed where they are sympatric, these barriers could play a role in the maintenance of reproductive isolation between them. The number of eggs that fail to develop represents a substantial cost of hybridization to *G. bimaculatus* females, and this cost could reinforce the evolution of barriers occurring earlier in the reproductive process.

## Background

It has long been recognised that the evolution and maintenance of new species requires reproductive isolation, whereby barriers to interbreeding prevent gene flow between incipient species [1,2]. The nature of these barriers, and their evolution, has been a central focus of speciation research. They have been traditionally studied in terms of those occurring prior to mating, or those that affect hybrid fitness [3], with measures taken from observable offspring, or inferred from reduced offspring counts. Less attention has been paid to barriers occurring within the female or within the egg, presumably because of the difficulty of studying these mechanisms. New techniques are now allowing important insights to be gained, and there is increasing interest in how these difficult to observe post-mating mechanisms might act to maintain reproductive isolation (for example [4-6]).

Instances of mixed-species pairings producing fewer offspring than pure-species pairings have been recorded in a number of species, and are often attributed to differences in uptake and storage of sperm [7,8], or the capacity of sperm to reach and fertilise eggs [9,10]. If these cryptic processes are successful, then the production of fewer hybrid offspring may be due to intrinsic inviability causing arrest during embryogenesis. Although the genetics of hybrid inviability have been studied extensively (reviewed by [11]), there are few studies of animal species in which reduced reproductive output is directly attributed to embryonic mortality. Even so, reports of this phenomenon come from a broad range of taxa [12-14], suggesting that embryonic mortality may be a common feature of hybridising systems.

Here we aim to determine whether there are barriers occurring prior to and during embryogenesis in the hybridising field crickets *Gryllus bimaculatus* and *G. campestris*. These sister species [15] have overlapping distributions through central Spain [16,17], and potentially further East [18]. Their evolutionary history is unknown, though we speculate that they may have diverged in allopatry, and have since come back into contact. The extent of contact and interbreeding between the species in the wild is unknown. In the laboratory *G. campestris* females almost never interbreed, whereas *G. bimaculatus* females hybridise readily with *G. campestris* males [19,20]. Female *G. bimaculatus* respond readily to mating signals from male *G. campestris* and hybrid offspring are both viable and fertile [19]. The lack of premating barriers suggests that, if individuals of these species frequently encounter one another in the areas where they coexist, post-mating barriers may play a role in the maintenance of reproductive isolation between them.

Prior to mating, a male provisions a spermatophore with sperm. After the male successfully attracts a female, the female mounts the male, and the spermatophore is attached to her reproductive tract. Sperm then transfer into the female, and are stored in the sperm storage organ, or spermatheca. The uptake and storage of sperm has been shown to be a significant barrier to interbreeding in this system. A recent study identified strong conspecific sperm precedence, whereby *G. bimaculatus* females doubly mated to both *G. bimaculatus* and *G. campestris* males preferentially stored sperm from the conspecific male [21]. The spermatheca in this species is approximately spherical, a shape which is likely to promote sperm mixing rather than stratified storage [22,23]. The representation of a male in the spermatheca is therefore expected to directly predict his success in siring offspring [24]. However this relationship did not hold. Conspecific males sired an even greater proportion of offspring than predicted from patterns of sperm storage indicating the presence of additional post-mating barriers to hybridisation. Here we use a fluorescent staining technique to identify when these subsequent barriers occur. We firstly assess whether eggs from interspecies pairings are less likely to be fertilised and start developing, and secondly assess whether developing embryos arrest before hatching. We aim to establish the potential for fertilisation and embryogenesis to act as barriers to interbreeding, providing insights into the mechanisms of reproductive isolation, and demonstrating that multiple post-mating barriers to interbreeding may be present between these species. These insights are likely to be applicable to a broad range of species, and may encourage comprehensive studies of post-mating barriers in other hybridising species.

## Results

### Early stage embryogenesis

There were three cases of *G. campestris* males failing to produce any normally developing eggs when mated to either a heterospecific or conspecific female. These cases were assumed to be due to infertility, and any data associated with these individuals were removed from the dataset prior to analyses. Of the remaining females that laid eggs, 13 were in BB mating pairs, 16 in BC mating pairs, and 16 in CC mating pairs.

**Figure 1:**
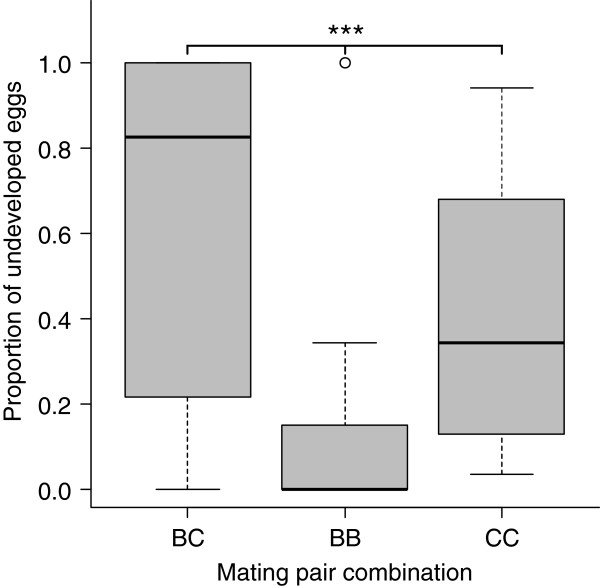
**The proportion of eggs that failed to develop across the mating pair types. **In each combination, female species is denoted by the first letter, and male species by the second. Data are displayed as medians (thickened line) and inter-quartile ranges (grey boxes), circles are outlying values. There are significant differences between all mating pair combinations. Statistical significance from glm (see text): *** *P* < 0.001.

**Figure 2:**
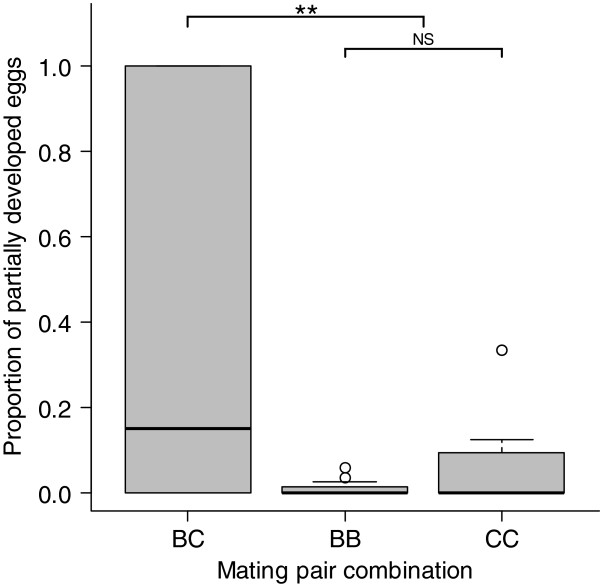
**The proportion of eggs that only partially developed across the mating pair types. **In each combination, female species is denoted by the first letter, and male species by the second. Data are displayed as medians (thickened line) and inter-quartile ranges (grey boxes), circles are outlying values. While BB and CC mating pairs do not differ in the proportion of eggs that only partially develop, heterospecific (BC) mating pairs differ from the conspecific (BB & CC) mating pairs. Statistical significance: NS *P* > 0.05; ** *P* < 0.01.

**Figure 3:**
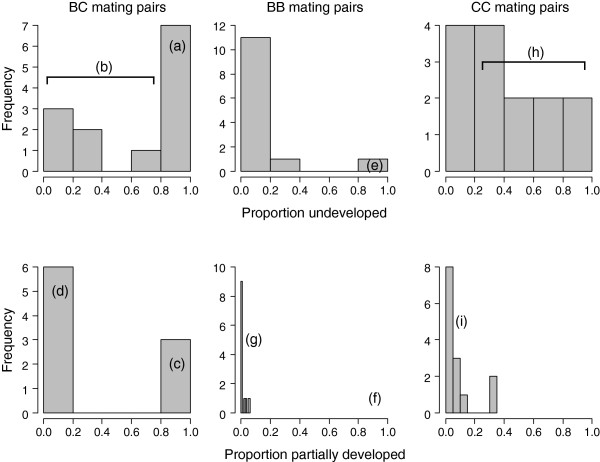
**Frequency histograms of egg development across mating pair combinations. **Histograms showing the frequency distributions of the proportion of eggs that did not develop (upper row), and the proportion of partially developed eggs (lower row), across each mating pair combination (by column left to right; BC, BB and CC). For BC mating pairs, more than half the clutches had a majority of undeveloped eggs (**a**). The remaining clutches varied in the proportion of undeveloped eggs and eggs beginning embryogenesis (**b**). Of these clutches showing signs of development, there was a bimodal distribution of either a majority of eggs partially developing (**c**), or eggs developing normally (**d**), rather than a normal distribution of development success. BB clutches rarely consisted of undeveloped eggs (**e**) or partially developed eggs (**f**). Instead, clutches had a majority of eggs that developed normally (**g**). The majority of CC clutches consisted of at least some undeveloped eggs (**h**). Of the eggs beginning embryogenesis, almost all developed normally (**i**).

The proportion of eggs failing to develop differed among the mating pair combinations (lmer; χ ^2^_2,4_ = 19.11, *P* < 0.0001, Figure 1), with *G. bimaculatus* females mated to heterospecific males (BC) laying the greatest number of undeveloped eggs, and *G. bimaculatus* females mated to conspecific males (BB) laying the fewest. Of the eggs that started to develop, the proportion that only partially developed differed significantly among the mating pair combinations (lmer; χ ^2^_2,4_ = 12.14, *P* = 0.0023). Post hoc tests revealed that this difference was due to eggs from heterospecific pairings (BC) being more likely to only partially develop than eggs from the conspecific pairs (BB & CC) (lmer; χ ^2^_1,3_ = 9.75, *P =* 0.0018*,* Figure 2). There was no difference between the conspecific pairs (BB & CC) in the proportion of eggs that only partially developed (lmer; χ ^2^_1,4_ = 2.39, *P* = 0.122). The cases of eggs only partially developing tended to be concentrated within a few clutches, rather than being equally spread across clutches (Figure 3, see (c) and (d)).

### Late stage embryogenesis

**Figure 4:**
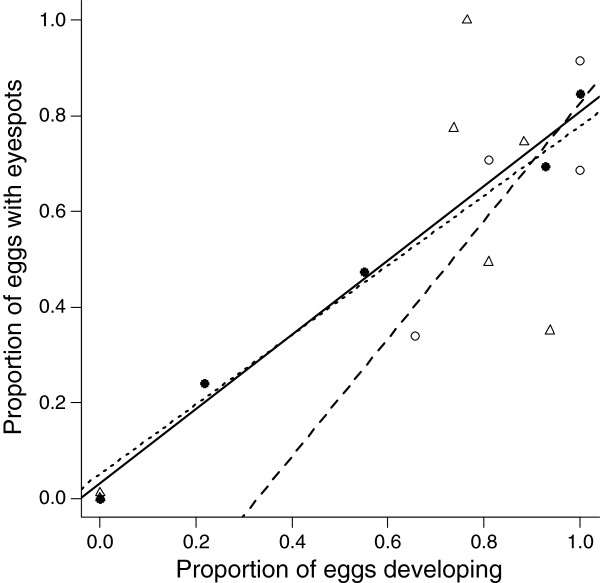
**The relationship between early and late stage embryogenesis. **The proportion of eggs developing normally at early embryogenesis predicts the proportion that contained late stage embryos with eyespots. Clutches from BC mating pairs are shown by closed dots and solid line. BB mating pairs are shown by open dots and broken line. CC pairs are shown by open triangles and dotted line.

**Figure 5:**
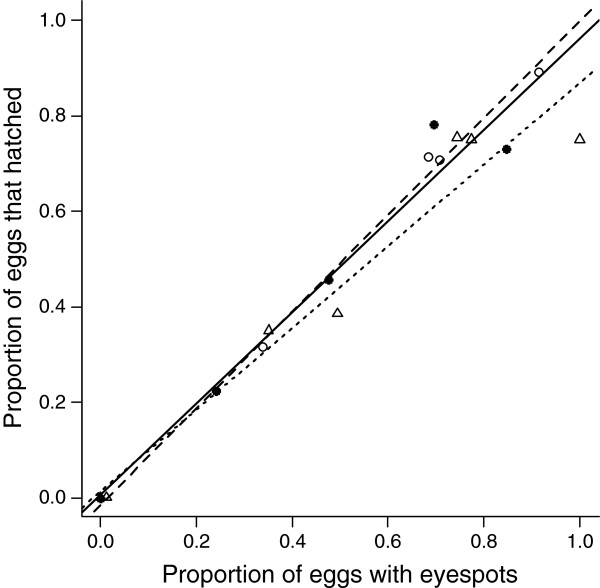
**The relationship between late stage embryogenesis and hatching success. **The proportion of eggs containing embryos with eyespots predicts the proportion that subsequently hatched. Clutches from BC mating pairs are shown by closed dots and solid line. BB mating pairs are shown by open dots and broken line. CC pairs are shown by open triangles and dotted line.

Among clutches, the proportion of eggs that showed successful early development predicted the proportion of eggs that contained late stage embryos with eyespots (glm; F_1,14_ = 25.31, *P* < 0.001). This relationship did not differ among the mating pair combinations (glm; F_2,13_ = 0.31, *P* = 0.740, Figure 4). Almost all eggs containing late stage embryos with eyespots went on to hatch (glm; F_1,14_ = 193.65, *P* < 0.001), and likelihood of death did not differ among the mating pair combinations (glm; F_2,13_ = 0.33, *P* = 0.726, Figure 5).

## Discussion

There are multiple post-mating barriers to hybridisation between these species, which may act to maintain reproductive isolation if individuals meet and mate in the wild. A strong isolating mechanism at the stage of uptake and storage of sperm has already been demonstrated, and the inferred presence of further mechanisms between sperm storage and the hatching of eggs highlighted [21]. Here we show that another potential barrier occurs between sperm storage and mitosis within the egg, and another postzygotic barrier during early embryogenesis, the pattern of which indicates the existence of polymorphism in hybrid incompatibility.

Eggs from mixed-species pairs were far less likely to begin embryogenesis than those from pure-species pairs. This has likewise been demonstrated in other interbreeding species. Almost all eggs from heterospecific crosses between *Gryllus firmus* and *G. pensylvanicus* go unfertilised [6]. Similarly, almost all eggs laid by *Drosophila virilus* females mated to *D. novamexicana* are not fertilised [5]. Fewer eggs hatch from mixed species pairings of *Drosophila santomea* and *D. yakuba* than from pure-species pairings, assumed to be due to lower rates of fertilisation. There are a range of mechanisms between storage of sperm and fertilisation that could be driving this barrier to interbreeding, since interactions between sperm and females are complex. For example, heterospecific sperm may have reduced survival in the spermatheca [8], or may be less able to traverse the female’s reproductive tract [9]. Upon reaching the site of fertilisation, the sperm may be unable to penetrate the egg wall [25], or the eggs may be incorrectly fertilised [26]. If fertilisation is successful, then development may fail very early on, prior to the onset of mitosis. Our study does not allow us to resolve which of the cascade of sperm-female interactions, sperm-egg interactions, or early developmental failures act as isolating mechanisms in the *G. bimaculatus* and *G. campestris* system, however it is likely that a combination of factors are involved, as has been demonstrated in *Drosophila melanogaster*[27].

In our system we found a large number of eggs from mixed-species pairs that failed to develop, which, notably, were not equally spread across clutches. While some clutches were dominated by eggs that showed no signs of embryogenesis, in others almost all eggs started developing. The cases of entire clutches failing cannot be explained by male infertility, since this was ruled out in our methodology. Instead this may be due to differences among individuals in traits that affect fertilisation success, driving incompatibilities between particular mating partners. If there are individual differences in the traits of sperm, then we might expect variation among males in the success of sperm-female or sperm-egg interactions. In cases of heterospecific crosses, this variation might translate into extreme differences in individual success, whereby the traits exhibited by some males are complementary to those of the female, whereas others are not. This could produce the binary success and failure of hybrid clutch development observed in this study. More likely, the pattern we observe represents an example of variable reproductive isolation (VRI) [28], driven by intrinsic post-zygotic inviability. Alleles from different species can interact negatively when brought together in hybrids, reducing their fitness in terms of viability or sterility [29-31]. Rather than the traditionally held assumption that the alleles involved in these incompatibilities are fixed within species, they could instead be polymorphic, contributing to intraspecific variation in the extent of reduced hybrid fitness. Interest in this variation is growing, and there is now a modest list of examples in the literature (reviewed in [28]). In animals, these examples come from a range of species, including frogs [32], fish [33], grasshoppers [34], flies [35], and mice [36]. There are important insights to be gained about the process of speciation from studies of VRI, and explicit investigation of this in *G. bimaculatus* and *G. campestris* would provide a valuable contribution to this emerging field.

We would expect the proportion of eggs that failed to develop to be similar between the pure-bred *G. campestris* eggs and the pure-bred *G. bimaculatus* eggs, however we found a significant difference between these groups. While this might be explained by differences between the species in egg viability, it is perhaps more likely due to difference in responses to the laboratory environment. In the wild *G. bimaculatus* are found in hot, arid environments, whereas *G. campestris* are found in more temperate regions [17]. The relatively high temperature maintained in the laboratory may therefore be sub-optimal for egg development of *G. campestris*. Furthermore, unlike the *G. campestris* which were wild-caught, our *G. bimaculatus* crickets had been reared in the laboratory for a number of generations, so might possibly have become somewhat lab adapted. This likely difference in the optimum temperature for egg incubation might have also had an influence on the developmental success of hybrids, but the existence of differences between the hybrid eggs and eggs from both conspecific pairings demonstrates that their low success is not just a temperature artefact.

As well as the strong barrier prior to embryogenesis (≤4 nuclei), we have also identified a difference in developmental success after the onset of mitosis. Eggs were all a minimum of 10 hours post-laying, by which time the nuclei of a developing egg would be expected to be uniformly distributed over the surface, soon to form the blastoderm [37]. Of the eggs that began embryogenesis, we found that those fertilised by heterospecific sperm were more likely to arrest during early development. Most of these partially developed eggs contained fewer than 20 nuclei, suggesting that arrest occurred within the first few mitotic divisions, long before blastoderm formation. We found no evidence for a barrier late in development - the relationship between the number of eggs with eyespots and the number that successfully hatched was strong, regardless of the species identities of the parents.

While we find hybrid arrest in field crickets occurring during very early development, prior to blastoderm formation, the few examples in the literature from other animal species report a range of stages at which arrest may occur, notably around the time of gastrulation, the stage at which three distinct germ layers are formed. Hybrid eggs laid by female *Drosophila pseudoobscura* mated to male *D. miranda* degenerate within a few hours of fertilisiation [38], and likewise eggs from crosses between *D. virilis* and *D. littoralis* arrest during the first few divisions of cells [39]. Hybrid eggs produced by females of the common duck, *Anas platyrhnchos*, inseminated by the Muscovy duck, *Cairina moschata*, are likely to arrest early in development, prior to blastoderm formation [14]. Crosses in which the eggs of the sea urchin *Heliocidaris tuberculata* are fertilised by *H. erythrogramma* sperm result in arrest at gastrulation, due to differences between the parental species in how axes of asymmetry are determined [40]. Among a number of the nematode genus *Caenorhabditis*, hybrid embryos arrest due to defects in the initiation of gastrulation, or later, during compaction or elongation of the embryo [41]. The hybrid embryos produced by female *Rana catesbeiana* and male *R. clamitans* frogs develop an abnormal elongated gastrula, and are unable to develop further [42]. In hybrid toads, abnormalities occur later in development, with embryos from crosses between female *Bufo fowleri* and male *B. americanus* often failing during body elongation and development of the tail bud [43]. Hybrids between female brown trout, *Salmo trutta*, and male Atlantic salmon*, S. salar*, die even later in development, mainly between hatching and complete yolk absorption [13]. Hybrids between populations of *Podisma pedestris* grasshoppers cease to develop at a range of embryonic stages [44]. Likewise, embryos from crosses between five lamprey species vary in the stages at which fatality occurs, ranging from four cells, through to the hatching of larvae. The stage of fatality depends upon the parental species, occurring earlier with increasing genetic distance between dam and sire [45].

Although the genetics of hybrid inviability have been well studied (for example [46-49]), many of these studies refer vaguely to ‘hybrid lethality’ without verifying when this occurs. And despite the widely recognised importance of studies of hybrid embryos in the field of developmental biology, there has been surprisingly little attention paid in the context of reproductive isolation, with only a handful of reports of failed embryogenesis in hybrid animals. Despite this, reports come from a broad range of taxa, suggesting this may be a common phenomenon in hybridising systems. As well as acting at a variety of stages among species, arrest is sometimes unidirectional, only affecting one cross, and often only affecting one of the sexes. This indicates there is no common underlying mechanism to hybrid embryo mortality, and has led to a number of genetic modes being implicated [11].

## Conclusions

We have demonstrated that there are multiple mechanisms occurring after sperm storage that reduce the reproductive success of crosses between *G. bimaculatus* females and *G. campestris* males. Eggs from this heterospecific cross were less likely to begin embryogenesis, and if they did begin developing, they were more likely to arrest than eggs from conspecific mating pairs. There was bimodal variation among hybrid clutches in the number of developing embryos that underwent arrest, suggesting that this is an example of variable reproductive isolation driven by polymorphic genetic incompatibilities. If *G. bimaculatus* and *G. campestris* attempt to hybridise in the wild, these post-storage barriers have the potential to be important in the maintenance of reproductive isolation between them, and may have also played a historical role in the initial divergence of the populations. The potential for these mechanisms to reduce gene flow might even be reinforced by selection against hybridsation as they create a cost to interbreeding. Despite the viability and fertility of hybrids that do hatch in this system [19], the number of eggs that fail to develop represent a substantial cost to *G. bimaculatus* females. Eggs are energetically expensive to produce, and so females should avoid laying clutches of eggs that don’t yield offspring, and thus avoid interbreeding. This cost could reinforce the evolution of barriers occurring earlier in the reproductive process.

## Methods

*G. bimaculatus* were collected from Valencia, Spain in 2011 and reared in the lab for ~4 generations. Crickets were housed at 28°C under a 16:8 light:dark cycle, with food and water provided *ad libitum*. Last instar nymphs were isolated to ensure virginity upon adult emergence. *G. campestris* were collected near Gijon, Spain in spring 2012 as last instar nymphs or adults. These wild caught individuals were kept in the laboratory for at least 7 days prior to use in trials. All individuals were a minimum of 7 days old post-emergence before use in experimental trials to ensure sexual maturity.

### Matings and oviposition

Prior to heterospecific mating trials, males were exposed to non-experimental conspecific females to stimulate spermatophore production and to encourage courtship behaviour. These stimulating females were separated from the males by wire mesh so that the female could be detected, but not mated with. Virgin *G. bimaculatus* females were paired with either a conspecific (BB pairing) or a heterospecific (BC pairing) male. Those *G. campestris* males successfully mated to a heterospecific female were subsequently mated to a conspecific female (CC pairing), to confirm that any failure to fertilise *G. bimaculatus* eggs was due to post-mating reproductive barriers rather than infertility. Virgin *G. campestris* females were only paired with conspecific males as they will almost never interbreed [19,20,50]. Mating pairs were placed in a 11 × 11 cm arena lined with paper for traction and observed. If courtship or mating did not occur within approximately 1 h the male was replaced with another or trialled on subsequent days. Successful mating was confirmed by the presence of a spermatophore attached to the female. Post-mating, the pair were left in the arena for around 1 h, the time required for most of the spermatophore contents to be taken-up by the female [22].

Females were then housed individually, and provided with a small dish of damp sand to oviposit in for ~48 h. These dishes were replaced at intervals so that each female was provided with 4 dishes over the ~48 h period. After removal from the female, dishes were incubated at 28°C for a minimum of 10 h, up to ~24 h, before being processed. Eggs were then removed from the sand and counted. If fewer than 20 eggs were laid in each dish then all were processed for assessment of early stage embryogenesis. If a large number of eggs were laid, then 20 were randomly selected for processing, and the rest were incubated on damp cotton wool to assess late stage embryogenesis and hatching.

### Assessment of early stage embryogenesis

Soon after laying (within 3 h), a fertilised egg begins meiosis, and divisions can be seen as a female pronucleus and polar bodies (≤4 nuclei) on the dorsal side of the egg. The pronucleus then migrates to the ventral side of the egg where sperm enter through micropyles. Here, male and female pronuclei fuse, and mitotic division begins [51]. After 9 h, more than 100 nuclei can be seen on the surface of the egg, and after 12 h, around 500 nuclei will be uniformly distributed on the surface, forming the blastoderm [37]. Unfertilised eggs will often undergo initial meiotic division, but will never progress to have more than 4 nuclei [52]. Without sampling eggs within 2 min of laying when sperm might still be seen [37], it is not possible to tell whether eggs that only ever have ≤4 nuclei have not been penetrated by sperm, whether fusion between gametes has not occurred, or they were fertilised but development has arrested before the onset of mitosis. In attempting to assess this we might risk missing the sperm, leading us to draw false conclusions about fertilisation success. In addition to this, regular disturbance of females deters them from ovipositing, further hindering the ability to assess fertilisation in newly laid eggs. We therefore made no attempt to investigate this, and instead categorised any egg with ≤4 nuclei as ‘undeveloped’, while any egg with more than 4 nuclei was considered to have started embryogenesis [4,6]. Since we processed eggs after a minimum development time of 10 h, we conservatively expected at least 100 nuclei to be seen if an egg were developing normally, or fewer if embryogenesis had started and subsequently arrested. These were categorised as ‘normally developing’ (>100) and ‘partially developed’ (5–100), respectively.

**Figure 6:**
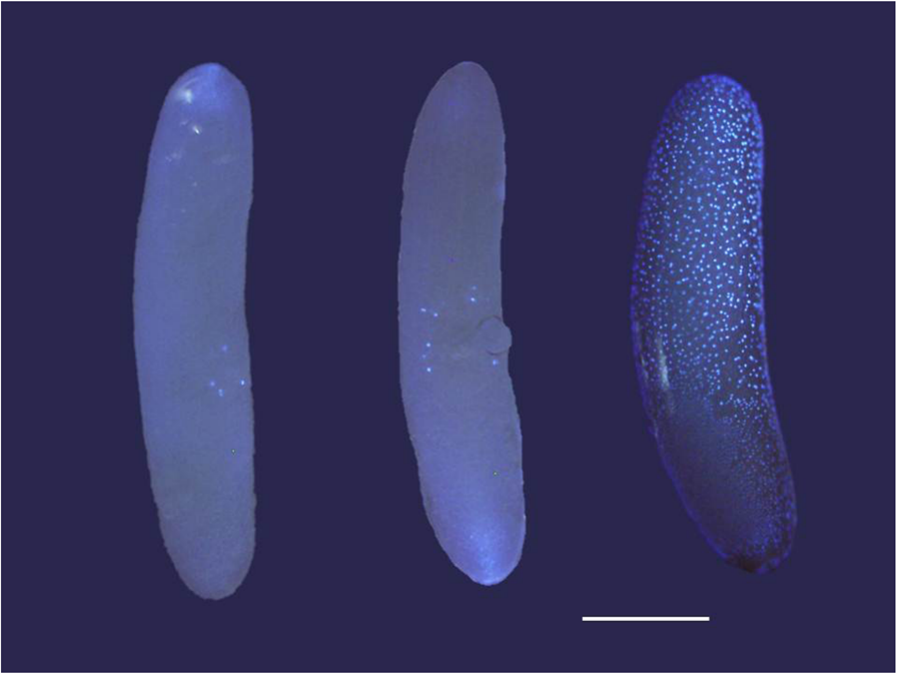
**Examples of eggs stained with DAPI, all ~48 h post laying. **From left to right: undeveloped, partially developed and normally developing eggs. Bar = 0.5 mm.

The protocol for preparing and staining eggs is adapted from the methodologies of Sarashina *et al*. [37] and Larson *et al*. [6]. To remove the thick opaque chorion, the eggs were firstly soaked in 50% bleach for 5 min at 22°C and gently shaken. They were then washed 3 times with phosphate buffered saline (PBS) solution, and fixed in equal parts paraformaldehyde (4% in PBS) and heptane for 20 min at 22°C, with gentle shaking. Eggs were washed again and then stored in methanol at 4°C until staining. Eggs were stained with 4’ , 6-diamidino-2-phenylindole (DAPI) for 20 min at 22°C with gentle shaking, then transferred to a microscope slide and viewed using a fluorescent microscope (Olympus BX61) and analySIS^D^ software. Each egg was visually inspected for nuclei, seen as fluorescent blue dots, and categorised as undeveloped (≤4), partially developed (5–100), or normally developing (≥100) depending on the number of nuclei seen (Figure 6). Any captured images were colour and contrast optimised in analySIS^D^. Figure 6 was created by cropping 3 separate images and placing them alongside each other using Microsoft PowerPoint software.

### Assessment of late stage embryogenesis

If early stage embryogenesis is successful, then embryos will continue to grow, passing through several developmental stages until the embryo undergoes segmentation and the organs form distinct structures. At this stage the eyes of the embryo can be seen by the naked eye through the chorion. The eggs that were not processed for assessment of early stage embryogenesis were incubated on cotton wool, and after 8 days of incubation, the number of eggs in each clutch with visible eyespots was counted, as well as the number that subsequently hatched.

### Statistical analyses

To quantify early stage embryogenesis we calculated 3 proportions: ‘prop.undeveloped’; the proportion of all eggs that completely failed to develop (≤4 nuclei), ‘prop.partial’; of the eggs that began to develop, the proportion that only partially developed (5 – 100 nuclei), and ‘prop.developed’; the proportion of all eggs that continued to develop successfully (≥100 nuclei). To quantify late stage embryogenesis we calculated 2 more proportions: ‘prop.eyespots’; of the eggs incubated, the proportion that contained embryos with eyespots, and ‘prop.hatch’; of the eggs incubated the proportion that hatched.

### Early stage embryogenesis

The relationships between the mating pair combinations (BC, BB, or CC) and each of the proportions prop.undeveloped and prop.partial were analysed using generalized linear mixed models (lme4 package [53], R v 2.14.1 [54]), fitted with binomial error structures. The proportion of eggs (prop.undeveloped or prop.partial) was entered as the response variable. Mating pair combination was entered as the explanatory variable. Male ID, a unique number assigned to each individual, was entered as a random effect to control for multiple use of individuals. The overall significance of the explanatory term was determined through model comparison using likelihood ratio tests [55]. Any post-hoc comparisons between levels of the explanatory variable were likewise made through model comparison using likelihood ratio tests.

### Late stage embryogenesis

The relationship between the proportion of normally developing eggs at early embryogenesis (prop.developed) and the proportion of eggs that contained late stage embryos (prop.eyespots) was analysed using a generalized linear model. Data were overdispersed so the model was fitted with quasibinomial error structure. Prop.eyespots was fitted as the response variable. Prop.developed and the mating pair combination, as well as their interaction were entered as explanatory variables. The significance of the explanatory variables was determined through model comparison using F tests. The relationship between the proportion of eggs that contained embryos (prop.eyespots) and the proportion that subsequently hatched (prop.hatch), interacting with mating pair combination, was likewise analysed using a generalized linear model.

## Competing interests

The authors declare that they have no competing interests.

## Authors’ contributions

FT conceived of the study, carried out data collection, performed the statistical analyses and drafted the manuscript. TT participated in performing the statistical analyses, and RRM and TT helped to draft the manuscript and collected animals for the study. All authors read and approved the final manuscript.
